# Precision oncology: from large language models to multi-agent systems

**DOI:** 10.3389/fonc.2026.1828507

**Published:** 2026-05-04

**Authors:** Xiaotong Guo, Jun Chen, Yuye Zhang, Xinli Zhang, Jue Lu, Qian Qin, Jiaqi Chen, Xuan Gao, Jing Wang

**Affiliations:** 1 Department of Radiology, Union Hospital, Tongji Medical College, Huazhong University of Science and Technology, Wuhan, China; 2 Hubei Provincial Clinical Research Center for Precision Radiology & Interventional Medicine, Wuhan, China; 3 Hubei Key Laboratory of Molecular Imaging, Wuhan, China; 4 Bayer Healthcare, Wuhan, China; 5 Tongji Medical College of Huazhong University of Science and Technology, Wuhan, China

**Keywords:** AI, AI agent, large language model, multi-agent system, oncology

## Abstract

With the rapid growth of electronic health records, medical imaging, and high-throughput omics data, precision oncology faces increasing demands for cross-modal information integration and complex clinical decision support. In recent years, large language models (LLMs) and their multimodal extensions have opened new technological avenues for addressing these challenges and have shown considerable promise across a range of applications. This review provides a structured narrative overview of the current applications of these technologies across the precision oncology care continuum, encompassing key stages such as cancer screening, diagnosis, staging, treatment recommendation, and clinical documentation. However, single-model approaches still have clear limitations in constructing complex clinical reasoning pathways, ensuring the traceability and verifiability of decision processes, and integrating deeply with established clinical workflows. Against this backdrop, AI agents with autonomous planning and coordination capabilities, together with multi-agent systems (MAS), have emerged as an important new direction in precision oncology research. Building on this development, we further propose an analytical framework centered on task–architecture alignment, emphasizing that foundation models, single-agent systems, and multi-agent systems should be selected according to the complexity and risk profile of the clinical task. Such a framework may provide a useful basis for the design, evaluation, and clinical translation of AI systems in precision oncology.

## 1 Introduction

In recent years, large language models (LLMs) have been transitioning from single-task processors to multi-agent collaborative decision-making systems, a shift that could reshape precision-oncology care pathways (1–4). LLMs—originally centered on language understanding—have shown considerable promise for clinical text interpretation and knowledge integration, offering new tools to support clinical decision making. However, most extant models remain largely unimodal and text-centric and thus face clear limitations in complex clinical scenarios that require joint analysis of imaging, pathology, and other modalities. With the emergence of multimodal large language models (MLLMs), artificial intelligence has begun to acquire the capability to jointly process textual, visual, and structured information, bringing AI systems closer to the multimodal reasoning processes underlying real-world clinical decision making (5–7). Conventional LLM/MLLM architectures are typically configured to address isolated tasks and often lack the flexibility required by real-world clinical workflows. To address these shortcomings, AI agents and multi-agent systems (MAS) built upon LLMs have been proposed (8–10). AI agents possess autonomy, can leverage external tools, execute multi-step procedures, and coordinate with other agents. For example, without costly parameter updates, AI agents can connect to external knowledge sources to retrieve up-to-date guidelines and literature, integrating multidimensional evidence to produce personalized diagnostic and therapeutic suggestions.

With the maturation of multi-agent architectures, systems composed of multiple collaborating LLMs have attracted growing interest (11). In contrast to most existing large medical language models that operate under static, single-turn input settings, multi-agent frameworks incorporate LLM-based agents to more faithfully emulate the dynamic, iterative, and interdisciplinary interactions characteristic of real-world clinical practice. By assigning specialized agent roles and enabling multi-round deliberation, such systems can reduce information overload and cognitive burden while enhancing the robustness of clinical reasoning (12). Related studies have demonstrated promise in applications including Alzheimer’s disease prediction and neuro-oncology decision support (13–15). Existing reviews have examined this field from several complementary perspectives, including the broader evolution of generative AI in medicine, the general architectural paradigms of medical AI agents and multi-agent systems, the potential implications of AI agents for oncology, and the emerging roles of large language models and multimodal foundation models in precision oncology (2, 4, 5, 9, 11). However, an in-depth analysis grounded in specific oncological clinical tasks remains lacking, particularly one that clarifies the appropriate scope, functional roles, and limitations of different technical approaches across real-world oncology workflows. Against this backdrop, this Review synthesizes representative prior studies (**Supplementary Table 1**) and focuses on the full precision oncology care continuum. Across key stages, including screening, diagnosis, staging, treatment recommendation, prognostic assessment, and clinical text processing, we analyze the functional roles, applicability boundaries, and collaborative logic of foundation models, single-agent systems, and multi-agent systems. We further outline a layered collaborative framework based on task–architecture alignment, as a conceptual perspective for understanding how different AI paradigms may be appropriately integrated into oncology care workflows.

## 2 Literature search strategy

This study employed a structured literature search to identify and synthesize relevant studies on LLMs, MLLMs, AI agents, and MAS in precision oncology. The search strategy was developed in accordance with established recommendations for transparent reporting in narrative reviews, including the SANRA guidance (16).

Targeted searches were performed in PubMed, Google Scholar, and arXiv from January 2023 to January 2026. This time frame was chosen because studies directly related to LLMs, MLLMs, AI agents, and MAS in oncology have largely emerged within the past three years. Publications published before 2023, including selected studies from general medicine, preprints, and seminal methodological papers, were included only when they provided essential conceptual definitions, technical frameworks, or methodological background relevant to the present review. The search used combinations of the following terms and their variants: “large language model”, “multimodal large language model”, “AI agent”, “multi-agent system”, “agentic AI”, “ChatGPT”, “GPT-4”, “oncology”, “cancer”, and “precision oncology”.

Studies were included if they met the following criteria: first, they focused directly on oncology-related clinical tasks involving LLMs, MLLMs, AI agents, or MAS; second, when oncology-specific evidence was limited, a small number of representative studies from general medicine on AI agents or MAS were included to provide conceptual or methodological context; and third, eligible studies were required to have reasonably clear task definitions, methodological descriptions, and quantitative evaluation. Studies were excluded if they focused solely on AI architecture without a healthcare application or lacked substantive methodological or analytical contribution. In addition, the reference lists of key articles were manually screened to identify further relevant studies that might otherwise have been missed.

This review emphasizes representative evidence synthesis and clinical relevance rather than exhaustive inclusion of all potentially related publications. In total, 50 studies were included in the final qualitative synthesis.

## 3 Technological evolution and domain adaptation strategies

### 3.1 Technological evolution: from unimodal LLMs to multi-agent systems

**Figure 1:**
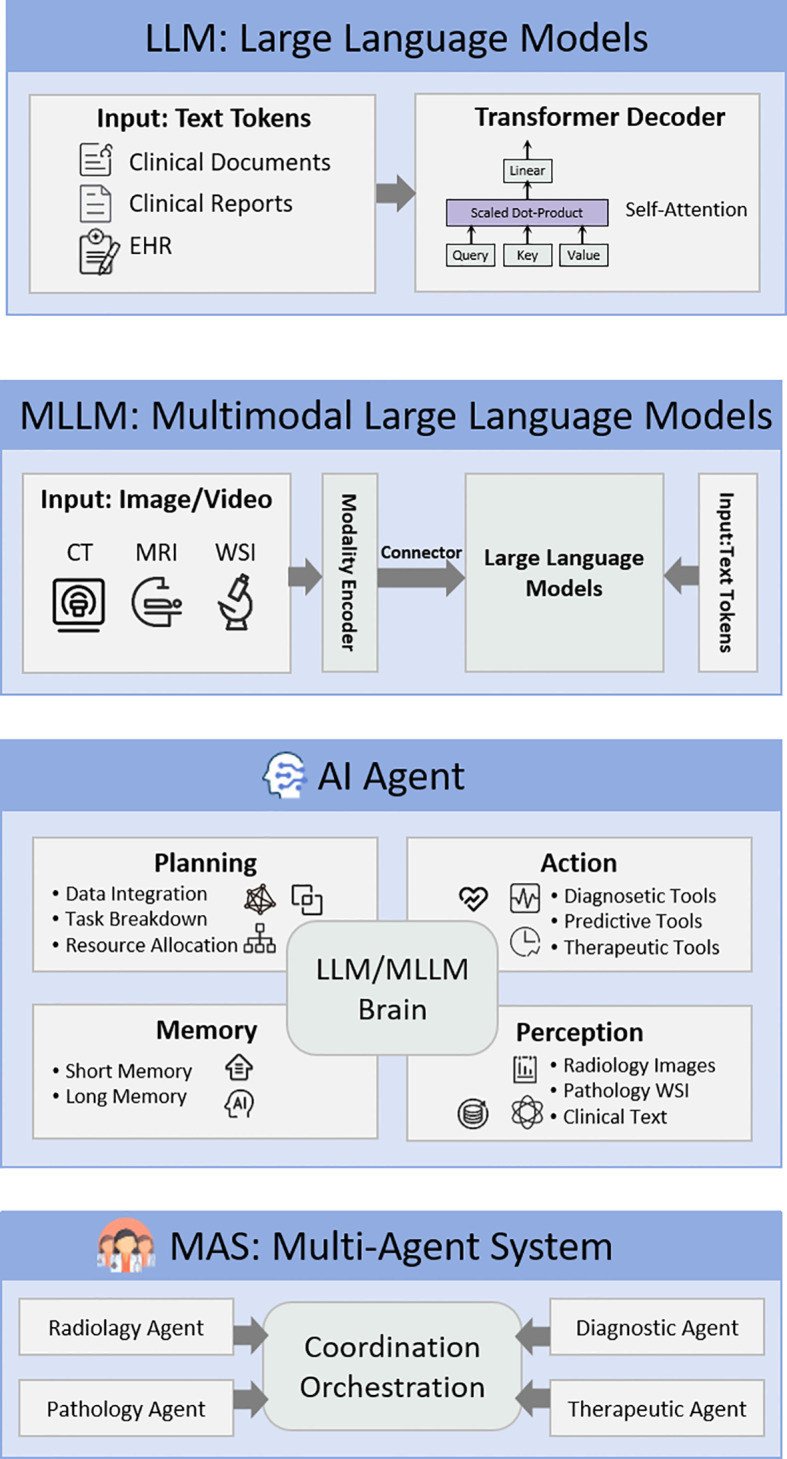
Hierarchical collaborative AI architecture for precision oncology. The framework progresses from foundational LLMs to MLLMs, and further to AI Agent equipped with planning, action, memory, and perception capabilities, culminating in MAS composed of multiple specialized agents collaborating via an orchestrator.

In this review, we categorize AI systems into four functional levels: (1) Foundation LLMs—focusing on textual understanding and reasoning; (2) Multimodal Large Language Models (MLLMs)—extending perceptual capabilities via visual or textual encoders; (3) Single-agent systems—autonomous units utilizing an LLM/MLLM as a cognitive core, endowed with planning, action, perception, and memory capabilities; and (4) MAS—system-level architectures composed of multiple specialized agents, coordination/orchestration layers, and tool management/auditing modules (**Figure 1**). This stratification helps delineate which architectural paradigm is best suited for specific clinical tasks.

LLMs are deep learning models based on the Transformer architecture, with billions of trainable parameters, which have undergone autoregressive pre-training on massive general corpora. Upon scaling, they exhibit emergent properties, such as zero-shot or few-shot reasoning. For these general-purpose LLMs, medical knowledge is primarily derived from the ingestion of biomedical texts during pre-training, the transfer of cross-task reasoning abilities, and subsequent instruction fine-tuning or retrieval-augmented strategies. These processes enable the models to master basic oncology terminology, diagnostic logic, and semantic structures, laying the foundation for precision oncology tasks (2, 17, 18). Some LLMs, even without specialized medical fine-tuning, have demonstrated the ability to accurately answer complex questions on medical examinations like the USMLE (19–21), notable examples include GPT-4, GPT-4o, and DeepSeek-R1 (22).

MLLMs build on LLMs by integrating the capability to receive, fuse, and comprehend heterogeneous information, such as images and text (23). Through cross-modal alignment and multimodal instruction fine-tuning, they enable them to handle complex “multi-input, multi-output” tasks (24). Typical representatives include GPT-4o, GPT-5 and Gemini 3 (25, 26). In oncology applications, multimodal models can integrate radiology images, pathology slides, and clinical reports (including both structured and unstructured information) to facilitate a more comprehensive understanding of patient conditions and support precision diagnostic decisions (17–19).

Despite the excellence of LLMs in specific tasks such as medical examinations and documentation (27, 28), and their emerging clinical utility (29), they face limitations in real-world oncology scenarios characterized by high complexity, multiple stages, and reliance on external tools and up-to-date knowledge. To address the challenges of highly complex, cross-disciplinary decision-making in oncology, researchers have proposed constructing medical AI agents that utilize LLMs/MLLMs as core reasoning modules equipped with capabilities for task planning, tool usage, perception, and memory (6, 30, 31). Furthermore, multiple agents with distinct specializations are organized into MAS via coordination mechanisms (32). This evolution marks a paradigm shift, transforming the system from a passive, reactive single-turn response model into an autonomous, collaborative Agentic AI (33).

**Figure 2:**
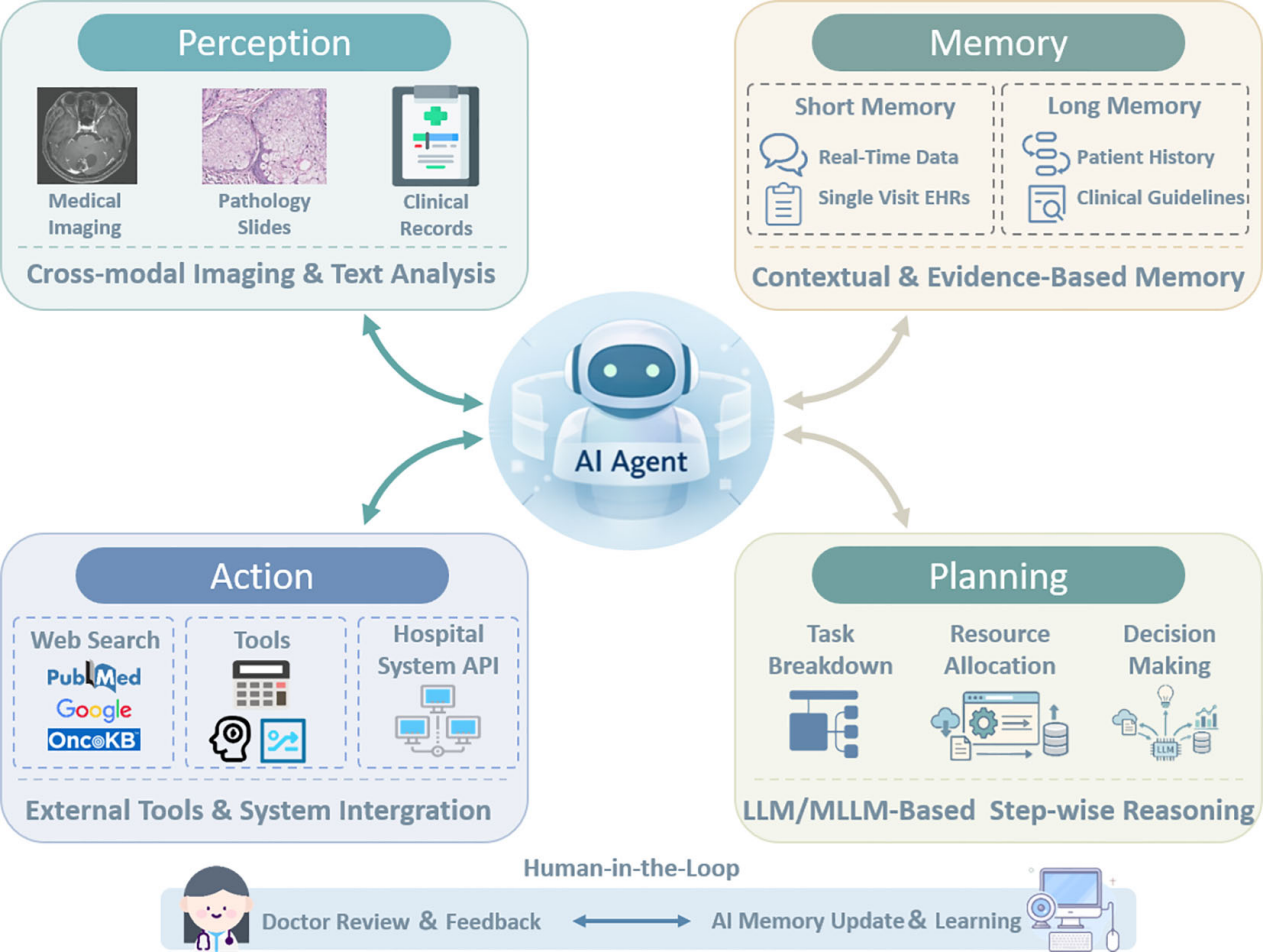
Architecture of an AI Agent–enabled clinical reasoning framework. The AI agent is organized around four functional modules: (1) Perception: Integrates medical imaging, pathology slides, and clinical records to enable cross-modal understanding of patient data. (2) Memory: Comprises short-term memory (session-level context, current clinical status of the patient) and long-term memory (longitudinal patient history, population-level evidence, and clinical guidelines), supporting evidence-based and context-aware reasoning. (3) Action: Enables interaction with external tools and systems, including biomedical databases (e.g., PubMed, OncoKB), analytical software, and hospital system APIs. (4) Planning: The core LLM/MLLM performs task decomposition, tool selection, and stepwise reasoning to generate interpretable clinical recommendations, such as diagnostic pathways, treatment planning, and follow-up strategies. Bidirectional information flow supports continuous interaction among modules, while a human feedback mechanism ensures clinician oversight and optimization. This architecture facilitates transparent, traceable AI-assisted decision-making, rather than fully replacing clinicians.

The AI agent is an intelligent system centered around a large language model (LLM/MLLM) capable of autonomously comprehending task objectives, performing planning, and executing multi-step operations, calling upon external tools when necessary to resolve complex problems (34, 35). By combining Retrieval-Augmented Generation (RAG), specialized tools, and external databases, an AI agent operates through a synergistic loop of planning, action, perception, and memory (36–38). Its advantage lies in extending single-step question-answering into automated, multi-step, traceable, and tool-integrated workflows (29–31). Its core value is breaking through the “functional isolation” and “static nature” of traditional medical AI, positioning it as an active collaborative partner in a realistic clinical workflow. It serves as an “individual computational unit” with autonomous decision-making capabilities, forming the fundamental building block of MAS (8) (**Figure 2**).

**Figure 3:**
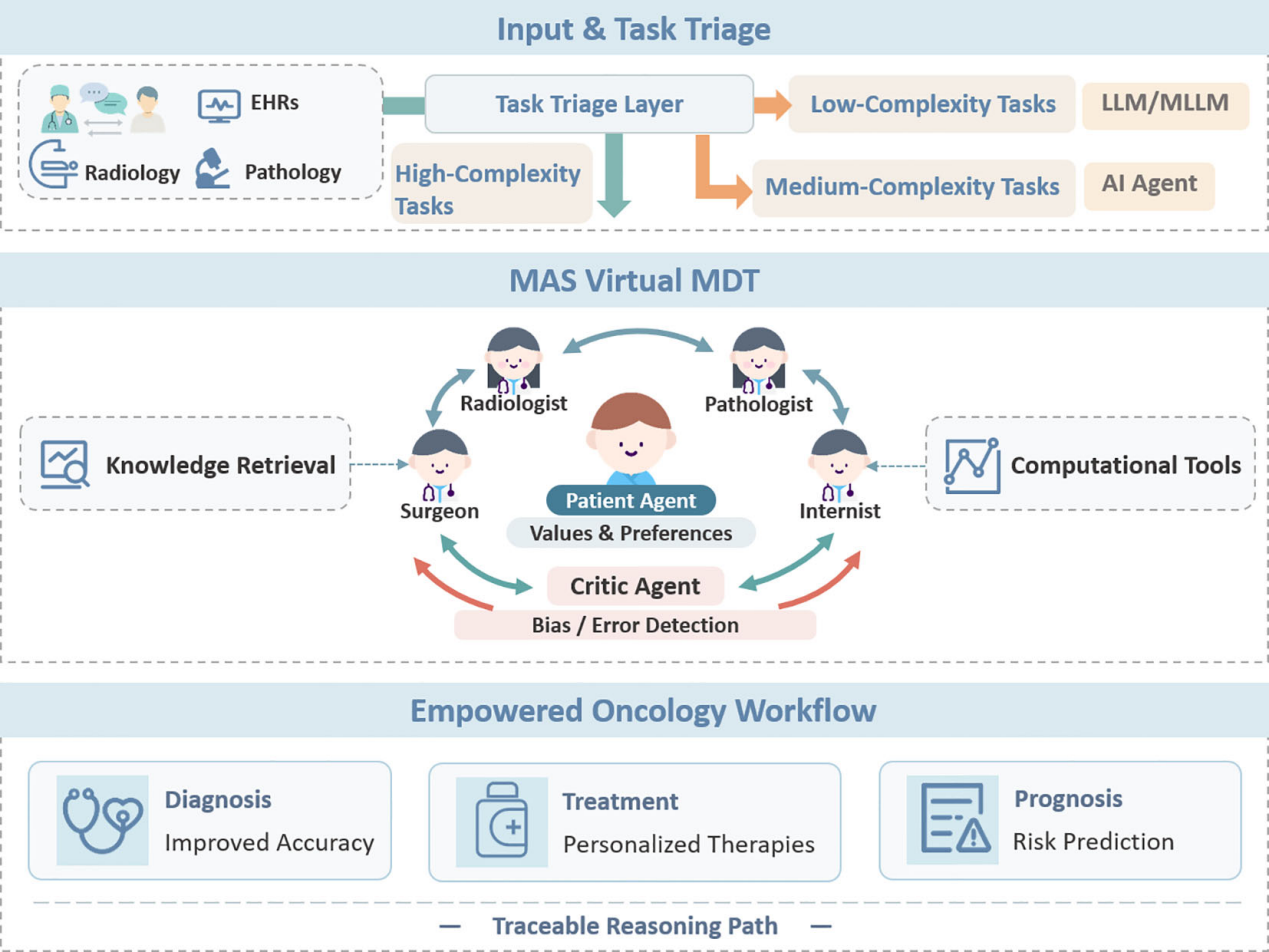
Multi-Agent System (MAS)-Empowered Oncology Workflow. Input & Task Triage: Multi-modal patient data, including EHRs, pathology, and medical imaging, are first organized through a qualitative task–architecture matching layer. Low-complexity tasks may be handled by LLMs/MLLMs; medium-complexity tasks may be supported by single-agent systems; and high-complexity tasks may require multi-agent systems. Core Process (MAS Virtual MDT): The MAS Virtual Multidisciplinary Team functions as the decision-making core, where specialized agents (e.g., radiology, pathology, internal medicine, surgery) collaborate through iterative reasoning. A Patient Proxy Agent represents patient values and preferences, while a Critic Agent monitors reasoning quality and mitigates potential bias. Output (Clinical Decision Support): The system supports improved diagnostic accuracy, personalized treatment planning (e.g., radiotherapy), and prognostic risk assessment. By providing a traceable reasoning pathway, the framework enhances transparency and trust in AI-assisted clinical decision-making.

MAS introduces a coordination mechanism on top of this foundation, enabling multiple agents with distinct expertise to divide labor and collaborate, akin to the Multidisciplinary Team (MDT) mode in clinical practice (39). For instance, a *Diagnostic Agent* interprets imaging and structured data, a *Retrieval Agent* accesses external databases for the latest evidence, a *Treatment Planning Agent* recommends regimens based on guidelines and individual risks, and a *Monitoring Agent* continuously parses disease progression. Together, they form a multi-role agent ecosystem tailored for the oncology care continuum (8) (**Figure 3**).

Preliminary studies indicate that when system architecture matches task complexity, agent-based systems can outperform baseline LLMs in some complex clinical tasks (8, 39). MAS provides a new mechanistic foundation for oncology research driven by cross-modal integration, long-chain reasoning, and dynamic feedback, enabling AI to not only propose treatment suggestions but also simulate the reasoning paths and strategies of clinical experts.

### 3.2 Domain adaptation strategies in LLMs

General-purpose LLMs often exhibit limitations in domain-specific knowledge and task alignment within oncology. To address these limitations, domain adaptation through fine-tuning has emerged as a central technical strategy. In essence, this approach further optimizes pretrained models using curated oncology-specific datasets, enabling them to retain general language capabilities while better aligning with the real-world oncology clinical setting. Core methodologies include supervised fine-tuning (SFT), reinforcement learning from human feedback (RLHF), and direct preference optimization (DPO). Furthermore, the integration of Low-Rank Adaptation (LoRA/QLoRA) techniques has significantly reduced training time and computational costs. Studies demonstrate that domain-tuned models significantly outperform their generic counterparts in oncology text extraction tasks (40), interpreting cancer-related clinical information with superior accuracy (41).

Beyond model fine-tuning, strategies such as prompt optimization, reasoning enhancement, and external knowledge integration can also improve the adaptability and accuracy of LLMs in clinical tasks, enabling rapid response to clinical needs without altering model parameters. In terms of strategy optimization, instruction fine-tuning and in-context learning significantly bolster model generalization. Specifically, Prompt Engineering refines task definitions to enhance information extraction precision (42). This few-shot or zero-shot learning paradigm confers a distinct advantage in cross-task transferability (43–45).

Regarding reasoning enhancement, the Chain-of-Thought (CoT) framework improves the model’s ability to tackle complex clinical problems—such as assessing resectability in pancreatic cancer—by making the reasoning process explicit (46). CoT also provides transparent reasoning paths, facilitating human-in-the-loop error localization and thereby enhancing model interpretability (42).

RAG addresses knowledge obsolescence and hallucination by dynamically querying external knowledge bases like PubMed and MIMIC-III (47). RAG supplements oncology-specific domain knowledge without the need for retraining and significantly improves the signal-to-noise ratio in long-text processing (40). Crucially, the outputs generated via RAG are **traceable**, enhancing the transparency of information extraction—a requisite for trustworthy decision-making in precision oncology (48).

## 4 Applications of LLMs and MLLMs across the precision oncology care continuum

### 4.1 Screening and risk assessment

Early cancer screening is paramount for improving patient survival rates. However, clinical implementation faces persistent hurdles, including suboptimal patient adherence, the complexity of dynamically evolving guidelines, and inter-observer variability in interpretation. Leveraging their extensive knowledge retention and textual reasoning capabilities, LLMs are emerging as transformative tools for screening and risk stratification.

One of the primary applications of LLMs in this context lies in the automation and standardization of imaging-based risk stratification. Standardized risk scoring systems developed by international, multidisciplinary expert panels underpin cancer screening and diagnostic workflows; however, manual interpretation of radiology reports remains susceptible to subjective variability. Recent studies have demonstrated that models such as DeepSeek-V3, GPT-4, and Qwen-7B-Chat can be applied to radiology reports for risk stratification in hepatocellular carcinoma (49), breast cancer (50), ovarian cancer (51), and prostate cancer (52), achieving performance comparable to that of experienced clinicians. However, the direct application of off-the-shelf models requires caution. Research assessing GPT-3.5, GPT-4, and Bard for BI-RADS classification based on MRI, mammography, and ultrasound reports revealed only moderate concordance with human readers, with a notable incidence of discrepancies that could lead to adverse changes in clinical management (53). To mitigate this, researchers have adopted hybrid strategies (51)—utilizing LLMs for key feature extraction coupled with deterministic algorithms for scoring—which improved O-RADS accuracy by circumventing the uncertainty inherent in complex model reasoning. Furthermore, techniques such as LoRA, knowledge-driven prompting, and multimodal fusion have significantly bolstered the precision and cross-center robustness of BI-RADS classification (50).

Beyond risk scoring, LLMs offer substantial value in enhancing screening adherence and reducing unnecessary biopsies. Evidence suggests that models like GPT-4 can synthesize patient history, endoscopy results, and prior records to generate screening or surveillance recommendations that align with clinical guidelines, achieving concordance rates in colorectal cancer that match or even exceed those of clinicians (54). Moreover, LLMs can translate complex medical procedures into patient-friendly language, thereby improving health literacy and compliance with screening protocols (55). In parallel, ThyGPT has been developed for ultrasound-based risk stratification of thyroid nodules, enabling a substantial reduction in biopsy rates without increasing the risk of missed diagnoses (56).

### 4.2 Diagnosis and classification

In the domain of tumor diagnosis and classification, the utility of LLMs is primarily predicated on their robust medical semantic understanding and capacity for generating structured output. Models such as ChatGPT-4o and DeepSeek-R1 have demonstrated the ability to accurately perform diagnosis and differential diagnosis of brain tumors based on outpatient clinical notes (57). Similarly, GPT-4 can accurately diagnose malignancy and determine tumor staging based on textual descriptions from pathology reports and ultrasound imaging (58, 59). The integration of Retrieval-Augmented Generation (RAG) strategies further enhances this capability; for instance, GPT-4o coupled with RAG achieved significantly higher accuracy in diagnosing neuropathological tumor subtypes from pathology reports (60). However, reliance solely on textual input has limitations. In tasks generating differential diagnoses from descriptions of skin tumor characteristics, ChatGPT-3.5 exhibited lower diagnostic accuracy, necessitating caution in its application (61). Conversely, open-source models like Mistral and Llama have been successfully utilized to extract key entities from pathology reports with high precision, automating AJCC staging and ATA risk stratification for thyroid cancer (62), thereby guiding macro-level therapeutic decision-making. In clinical practice, tumor diagnosis relies heavily on the in-depth analysis of pathology and radiology images, an area where generalist vision models and specialized MLLMs are deepening diagnostic precision. GPT-4o has demonstrated the capability to estimate the malignancy probability of lung nodules based on longitudinal CT imaging (63). Remarkably, GPT-4V, even without fine-tuning, can perform colorectal cancer histological subtyping, polyp identification, and breast cancer lymph node metastasis detection using only few-shot prompting. Its performance approaches or even surpasses that of task-specific deep neural networks, underscoring its potent generalization potential (64).

The emergence of MLLMs is shifting the paradigm of Whole Slide Image (WSI) analysis from separate convolutional neural network deployments to unified processing by vision-language models. Foundation models represented by TITAN (65) and PathChat (66) can directly integrate WSIs with clinical records. Through cross-modal alignment, these models accomplish histological subtyping, tumor grading, and even prognostic feature prediction. Studies indicate that TITAN achieves performance on WSI tasks comparable to or exceeding state-of-the-art models such as PRISM, GigaPath, and CHIEF (65), while boasting superior parameter efficiency. Crucially, TITAN supports cross-modal retrieval of similar rare cases, providing diagnostic decision support to pathologists and mitigating the risk of misdiagnosis. Nonetheless, generalist models exhibit instability in certain modalities; for example, in dermoscopic image evaluation, GPT-4V achieved a primary diagnostic accuracy of only 36% (67), highlighting the continued need for domain-specific optimization prior to clinical deployment.

### 4.3 Treatment recommendation

LLMs are progressively integrating into the core of oncological treatment decision-making, evolving from providing foundational consultation services with physician-level accuracy to functioning as comprehensive support systems that encompass surgical evaluation, precision drug matching, and radiotherapy planning. In the therapeutic domains of colorectal cancer, breast cancer, and oropharyngeal squamous cell carcinoma (OPSCC), LLM-driven chatbots have been demonstrated to provide medical information with an accuracy comparable to that of physicians (68–70). In an assessment of their capability to recommend diagnoses and treatment plans for renal cell carcinoma (RCC), AI-generated suggestions achieved an overall concordance of 62.1% with MDT decisions, indicating their potential to assist in RCC case evaluation and warranting further investigation (71). For surgical resectability decisions in pancreatic cancer, a research team utilized GPT-4 combined with CoT prompting, achieving 92% accuracy in classifying resectability from CT reports. This approach reduced the average review time by 42% compared to surgeons and significantly enhanced report readability (46).

To address the challenge of identifying targeted cancer therapies from the rapidly expanding medical literature, researchers developed Medical Evidence Retrieval and Data Integration for Tailored, a system based on Gemini Pro that employs RAG and CoT techniques (72). The model identified a broader range of therapeutic options than those proposed by a molecular tumor board. For the treatment of rare gynecological tumors (RGTs), a digital twin approach built on LLMs has been used to efficiently match patients with more precise, individualized treatment regimens based on biomarker analysis (73).

Moreover, MLLMs are also supporting the crucial task of delineating radiotherapy target volumes. In the field of radiation oncology for breast cancer, a multimodal AI model named LLMSeg, developed from Llama2-7B-chat and a 3D Residual U-Net, was created for the 3D context-aware automatic contouring of the Clinical Target Volume (CTV) (74). In the breast cancer scenario, it achieved Dice Similarity Coefficients ranging from 0.822 to 0.844 and also demonstrated advantages in prostate cancer scenarios, significantly outperforming traditional unimodal AI models. For head and neck tumors, another team developed Radformer, a vision-language model based on GPT-4 and a convolutional neural network (CNN), which outperformed the baseline 3D-UNETR model in the task of delineating tumor target volumes in patients (75).

### 4.4 Prognosis prediction

LLMs have emerged as a pivotal technology in the domain of cancer prognosis and risk stratification. In the realm of dermatology, a novel risk stratification system named AIRIS, built upon RAG technology, has demonstrated performance superior to existing standards for prognostic assessment in cutaneous squamous cell carcinoma (cSCC) (76). The Woollie model, built upon the open-source LLaMA architecture and refined through multi-stage medical fine-tuning, enables accurate interpretation of radiology reports and prediction of cancer progression. It demonstrates superior performance over general-purpose large language models across multiple tasks, supporting more efficient identification of disease progression in oncologic practice (77). Recent work has increasingly shifted toward multimodal fusion to capture complementary information. A Multimodal transformer with Unified maSKed modeling, known as MUSK, has been developed for precision oncology. It effectively integrates complementary information from pathology images and clinical reports. Evaluated on a cohort of over 8,000 patients, MUSK demonstrated robust predictive capabilities for clinical outcomes, including melanoma recurrence, pan-cancer prognosis, and immunotherapy response (78).

### 4.5 Text processing and structured extraction

The application of LLMs in oncology is evolving from simple text feature extraction to sophisticated longitudinal data integration and clinical decision support, demonstrating their potential to redefine medical text processing workflows. In radiology, GPT-4-based models have demonstrated exceptional capabilities for structured data extraction, capably mining high-quality features from CT reports across various malignancies, including lung (79), liver (80), and pancreatic cancers (81). By leveraging CoT prompting, one research team achieved an accuracy of 99.9% in pancreatic cancer feature extraction, significantly outperforming traditional surgical review processes while enhancing report readability and substantially reducing review time (46). Beyond single cross-sectional analyses, GPT-4 has also proven effective in integrating longitudinal MRI reports and clinical histories for glioma patients (82), generating concise, visually-oriented timelines of disease progression that provide a clear basis for multidisciplinary team discussions.

In contrast, pathology reports typically present greater textual complexity and verbosity. Nonetheless, studies have shown that GPT-3.5 can extract TNM staging, subtype classifications, and surgical margin status from pathology reports for lung cancer and pediatric osteosarcoma with high accuracy and F1 scores (83). In a real-world application for gynecologic oncology registries, both Gemini 1.5 and Qwen2.5 72B, using only prompt engineering, significantly surpassed manual registration accuracy for TNM stage extraction (84).

Notably, the choice of model strategy must be tailored to the specific characteristics of the text. While RAG can effectively boost the accuracy of extracting information like IDH mutation status from lengthy brain tumor pathology reports, it may conversely introduce noise and degrade performance when applied to shorter radiology reports (40). This finding highlights the need to tailor model strategies to specific tasks.

Furthermore, the application of LLMs has expanded to encompass broader clinical workflow optimization and privacy-preserving solutions. By accurately extracting key information from convoluted raw patient histories, GPT-4 can generate imaging requests of a quality superior to those manually drafted by physicians (85). Ambient AI, such as DAX Copilot, can automatically generate electronic health records (EHR) notes by recording clinical conversations via mobile applications (e.g., Epic Haiku), with pilot studies confirming its efficacy in alleviating physician burnout (86). To address data privacy concerns, locally deployed pipelines using open-source LLMs have been developed. These privacy-preserving solutions can automatically process diverse medical documents—including pathology reports, order forms, and treatment histories—to support large-scale data aggregation for clinical research and decision support (87).

Despite the immense potential of LLMs in oncological information extraction, persistent challenges include inferential errors, information omission, and the lack of standardized error evaluation frameworks. To address this deficit, one study developed an end-to-end LLM pipeline that incorporates flexible prompting templates and undergoes a rigorous, human-in-the-loop iterative refinement process guided by a comprehensive error ontology. This system achieved an average F1 score of 0.99 for extracting renal tumor subtypes and 0.97 for detecting metastases from 2,297 renal pathology reports using GPT-4. The framework has also been shown to be extensible to other malignancies, such as prostate and breast cancer (42).

## 5 Early explorations of AI agents in oncological practice

The clinical decision-making process in precision oncology is characterized by high complexity and multimodality, demanding the integration of diverse information sources, including pathology, radiology, genomics, and EHRs. In addition, such decision-making often necessitates the continuous retrieval of the latest evidence to support individualized diagnosis and treatment. Traditional medical AI models, typically designed for specific pre-defined tasks such as image classification or survival prediction, fall short in this context due to their lack of contextual awareness and autonomy. To address this gap, single-agent systems have been developed. These systems leverage LLMs as their cognitive core, enabling them to execute complex clinical workflows through mechanisms such as planning, action, memory, and perception (8). Single-agent systems have already demonstrated notable advantages in other domains requiring multi-step reasoning and planning (88, 89). Nevertheless, from the perspective of clinical translation, single-agent systems in oncology remain at a nascent stage (90).

Studies involving truly autonomous agents with both planning and closed-loop execution capabilities are still limited. At present, only one study by Ferber et al. provides direct evidence in support of this approach (91). In that work, the authors developed an autonomous AI agent based on GPT-4 and integrated it with MedSAM, a pathological image-based molecular feature prediction model, as well as external knowledge and retrieval tools such as OncoKB and PubMed to support multimodal decision-making in precision oncology. In an evaluation involving 20 multimodal patient cases, the agent achieved a tool-selection accuracy of 87.5%, increased the correctness of clinical conclusions to 91.0%, and improved response completeness from 30.3% with GPT-4 alone to 87.2%. These findings demonstrate the feasibility of transferring general-purpose LLMs to oncology-specific precision care through tool augmentation. However, the current level of evidence remains limited to a small-sample feasibility study. Robust support from multicenter, prospective studies embedded in real-world clinical workflows and using patient outcomes as endpoints is still lacking.

## 6 The current state of multi-agent systems in oncology

MAS, through role specialization, collaborative reasoning, and conflict coordination, are architecturally more aligned with the organizational logic of multidisciplinary team (MDT) care in oncology, and therefore may offer advantages in multimodal information integration, long-horizon decision-making, and high-uncertainty scenarios. However, current oncology-specific MAS studies remain highly heterogeneous in task design, data sources, and evaluation strategies, and the overall evidence base is still at an early stage. Whether MAS necessarily outperform well-designed single-agent systems in real-world clinical tasks still requires further empirical comparison (90).

MAS shows considerable potential in simulating oncology MDTs. At present, EvoMDT represents one of the more comprehensively evaluated oncology-specific studies (92). This system comprises four types of agents—diagnostic, therapeutic, safety-monitoring, and coordinator agents—and generates oncology treatment recommendations through structured knowledge retrieval and cross-agent consensus mechanisms. It was evaluated on six public oncology question-answering benchmarks and four real-world datasets involving breast cancer, lung adenocarcinoma, hepatocellular carcinoma, and lymphoma, with additional single-blind physician review. The results showed that its decision quality was comparable to that of human MDTs, while response time was reduced by approximately 30–40%. Its semantic consistency with expert plans, measured by BERTScore, ranged from 0.62 to 0.68, and it outperformed several leading foundation models in both guideline concordance and safety. The main limitation of this study is that it relied primarily on retrospective standardized case materials and still lacks prospective deployment and validation at the patient-outcome level.

In gastrointestinal oncology, a preprint study assigned endoscopic, textual, imaging, and laboratory information to dedicated agents and then used an MDT-Core agent to perform cross-modal integration and explicit conflict detection (93). Across 2,174 cases of gastric cancer, colorectal cancer, and esophageal cancer collected from multiple institutions, and evaluated in a blinded manner by three senior oncologists, the system achieved an overall score of 4.60/5.00, compared with 3.76/5.00 for the single-agent baseline. The improvements were mainly reflected in reasoning quality and medical accuracy, with fewer hallucinatory recommendations. In neuro-oncology, a conference abstract reported an agent-based modeling framework that incorporated the perspectives of different specialists together with patient preferences to evaluate feasible treatment pathways in the presence of value conflicts; in specific scenarios, its decisions showed high concordance with those of actual tumor boards (14).

Beyond MDT simulation, the value of MAS also lies in task decomposition and knowledge-enhanced collaborative reasoning, with current applications focusing on oncology-specific diagnosis and optimization of subspecialty workflows. For example, in lung cancer diagnosis, a preprint study reported that LungNoduleAgent decomposed pulmonary nodule diagnosis into three sequential modules: nodule detection, imaging description, and malignancy assessment, the last of which was completed through medical knowledge graph retrieval and multi-round inter-agent deliberation. Across two private datasets and the public LIDC-IDRI dataset, the system achieved malignancy classification accuracies of 86.7%, 81.2%, and 89.1%, respectively, substantially outperforming general-purpose models such as GPT-4o and several medical agent systems. The same study also reported that existing general medical agents achieved only 40–50% accuracy on lung cancer-specific tasks, compared with 75–80% on general medical tasks, suggesting the importance of disease-specific architecture design for oncology diagnostic applications (94). In radiation oncology, GPT-Plan implemented closed-loop optimization from parameter generation to dose evaluation through iterative collaboration between “dosimetrist” and “physicist” agents. A feasibility study showed that, for lung cancer and cervical cancer, the quality of IMRT and VMAT plans generated by this system was comparable to that produced by the ECHO automated planning method and expert planners (95). In supportive cancer care, OncoPainBot established a cancer pain management framework composed of four agent types—information extraction, pain mechanism reasoning, treatment planning, and safety checking—and completed retrospective validation on 516 real-world electronic medical records; its generated content showed high semantic consistency with clinical documentation, and its accuracy for analgesic recommendation was 0.841 (96). In pathology, PathFinder developed a multimodal multi-agent framework consisting of Triage, Navigation, Description, and Diagnosis agents for stepwise navigation, evidence collection, and integrated diagnosis on melanoma whole-slide images. It enabled sequential navigation and comprehensive diagnosis, reportedly improving melanoma classification performance by approximately 8% over the previous state of the art while also providing natural-language explanations (97). MELLMA, by contrast, used a framework combining multiple expert models with LLM-based arbitration to resolve disagreements among expert models on gastric and breast cancer pathology datasets; the authors reported that it outperformed individual CNNs, Transformers, and conventional ensemble methods (98).

## 7 Limitations and future outlook

Amidst the rapid evolution of artificial intelligence, precision oncology is undergoing a profound paradigm shift from ‘digitization’ to ‘intelligentization’. LLMs and MLLMs have demonstrated exceptional capabilities in processing vast quantities of heterogeneous medical data. Meanwhile, emerging AI agents and MAS have further empowered these systems with the capacity for autonomous planning and collaborative reasoning. However, this technological proliferation introduces a complexity of choice. The key to future advancements lies not in the blind pursuit of the largest models or the most intricate systems, but in constructing a hierarchical clinical ecosystem where the task–architecture alignment is meticulously matched to specific clinical task attributes, data characteristics, and multidimensional costs.

### 7.1 Future outlook: a hierarchical and collaborative hybrid intelligence ecosystem

In precision oncology, the choice of medical AI architecture should not default to greater autonomy or increasingly complex multi-agent collaboration. Rather, it should be determined primarily by the intrinsic complexity of the task, its risk level, and the degree of coordination it requires. Current studies and emerging clinical practice suggest that foundation LLM/MLLM systems, single-agent architectures, and multi-agent systems (MAS) each have distinct domains of applicability (99–101). This highlights the potential clinical value of a layered collaborative ecosystem built around task–architecture alignment.

In the framework proposed here (**Figure 3**), low-complexity tasks are typically characterized by clearly defined objectives, standardized workflows, relatively low risk, and limited dependence on multidisciplinary collaboration. Such tasks may often be addressed using LLM/MLLM-based systems and their extended variants, including standardized information extraction, patient education, structured report generation, and assistive pathology or imaging perception tasks. These applications rely primarily on the model’s capacities for language understanding, multimodal perception, and information integration, allowing foundation models to offer clear advantages in efficiency and deployment cost.

Tasks of intermediate complexity may involve limited multi-step reasoning, selective use of external knowledge, or relatively well-defined closed-loop workflows. In such scenarios, single-agent architectures may provide a pragmatic compromise between capability and implementation burden. Representative examples include guideline-based medication adjustment, recommendation of standard treatment regimens, follow-up suggestions for a single disease entity, and clinical decision-support tasks with explicit goals and decomposable steps. Compared with MAS, such architectures are more likely to achieve a pragmatic balance among performance, interpretability, and deployment cost, while also facilitating regulatory oversight and accountability attribution.

By contrast, high-complexity tasks involve substantial uncertainty, conflicting evidence, and high-risk decisions that require multidisciplinary collaboration. In such settings, MAS are more likely to demonstrate their distinctive value. Examples include individualized treatment trade-offs in rare malignancies, therapeutic pathway selection under conflicting evidence, and other high-stakes clinical decisions requiring input from multiple specialties. These scenarios are better suited to collaborative reasoning among multiple role-specific agents, which can approximate the deliberative process of an MDT.

### 7.2 Real-world challenges

Although this layered collaborative framework provides a valuable conceptual basis for AI deployment in precision oncology, its effective translation into routine clinical practice will require overcoming substantial challenges across technical development, governance and real-world implementation. Foundational models like LLMs and MLLMs have yet to fully overcome their propensity for “hallucinations” and overconfidence, potentially generating logically coherent but factually incorrect medical advice. Their “black-box” nature also poses significant challenges to interpretability, undermining physician trust. MLLMs encounter computational bottlenecks due to “token explosion” when processing volumetric data, such as CT and MRI scans, which poses resource constraints for practical deployment. The scarcity of high-quality, aligned image-text data also limits their spatial reasoning and small-lesion detection capabilities, which remain inferior to those of human experts (102, 103). Moreover, existing medical benchmarks fall short in three key dimensions: diversity of evaluation metrics, task definitions, and limited medical modality. Newer standards, such as the Feature-Oriented Radiology Task Evaluation (FORTE), have proven more effective than traditional metrics for evaluating MLLM performance in radiology tasks, highlighting the need for more comprehensive evaluation frameworks (104).

While single agents possess memory modules, their effectiveness is constrained by the context window of the underlying LLM, especially when processing longitudinal patient records spanning several years, which can potentially lead to the omission of critical treatment or allergy histories. The complex reasoning mechanisms of single agents can also introduce high latency, limiting their applicability in real-time clinical scenarios. From a regulatory perspective, existing frameworks (e.g., from the FDA) are primarily designed for single-task AI tools, necessitating the development of more comprehensive evaluation systems for autonomous agents (9).

MAS, through their orchestration mechanisms, coordinate multiple specialized agents, but this introduces what can be termed “compounded opacity” (99). A minor misjudgment by a single perception agent can be amplified through multiple rounds of negotiation, resulting in systemic diagnostic errors and making the decision-making process difficult to trace. This increases the burden of validation and regulatory compliance. Furthermore, MAS architectures typically entail manifold increases in computational and communication overhead, posing greater feasibility challenges for clinical deployment. Nevertheless, a recent general diagnostic benchmark study reported that MAI-DxO framework improved diagnostic accuracy while reducing cumulative testing costs under benchmark conditions, showcasing the potential advantages of MAS in the cost-benefit trade-off (105). Therefore, under current technological and regulatory conditions, MAS are best positioned as advanced consultation systems to assist expert decision-making, rather than as fully automated, patient-facing tools.

## 8 Conclusion

The evolution of artificial intelligence in precision oncology is unlikely to take the form of a single technology replacing all clinical tasks. Rather, it is more likely to emerge as a layered ecosystem of complementary architectures, each aligned with the demands of a specific clinical context. For standardized tasks involving information processing and perceptual support, LLMs and MLLMs have already demonstrated considerable efficiency and practical utility. For closed-loop workflows with clearly defined objectives and decomposable steps, single-agent systems may offer the most favorable balance between performance, interpretability and deployment cost. By contrast, for complex decisions characterized by high uncertainty, the need for multidisciplinary collaboration and the integration of heterogeneous sources of evidence, multi-agent systems represent a promising direction that warrants further investigation.

Looking ahead, progress in precision oncology will depend not on the indiscriminate escalation of model autonomy, but on the development of architecture selection strategies tailored to specific clinical problems, and grounded in task complexity, risk level and coordination requirements. Equally important will be the establishment of robust ethical oversight and carefully designed human–AI collaboration frameworks. Only under these conditions can AI move beyond its current role as an assistive tool for information processing and gradually evolve into a form of decision support that is sufficiently trustworthy, transparent and clinically governable for prudent use in oncology practice.

## Supplementary material

Supplementary Table 1 Representative studies on LLMs, MLLMs, AI agents, and multi-agent systems in precision oncology.
